# Development and Validation of the Middle Years Development Instrument (MDI): Assessing Children’s Well-Being and Assets across Multiple Contexts

**DOI:** 10.1007/s11205-012-0149-y

**Published:** 2012-10-02

**Authors:** Kimberly A. Schonert-Reichl, Martin Guhn, Anne M. Gadermann, Shelley Hymel, Lina Sweiss, Clyde Hertzman

**Affiliations:** 1 Department of Educational and Counselling Psychology, and Special Education, University of British Columbia, 2125 Main Mall, Vancouver, BC V6T 1Z4 Canada; 2 The Human Early Learning Partnership, University of British Columbia, Suite 440, 2206 East Mall, Vancouver, BC V6T 1Z3 Canada

**Keywords:** Middle Years Development Instrument (MDI), Children’s well-being, Contextual and social assets, Population assessment, Student self-report, Survey development, Psychometric validation, Knowledge-to-action, Community-school-research collaboration, Middle childhood and adolescence, Development, Health, Connectedness, School experiences, After-school time

## Abstract

Few instruments provide reliable and valid data on child well-being and contextual assets during middle childhood, using children as informants. The authors developed a population-level, self-report measure of school-aged children’s well-being and assets—the Middle Years Development Instrument (MDI)—and examined its reliability and validity. The MDI was designed to assess child well-being inside and outside of school on five dimensions: (1) Social and emotional development, (2) Connectedness to peers and to adults at school, at home, and in the neighborhood, (3) School experiences, (4) Physical health and well-being, and (5) Constructive use of time after school. This paper describes the theoretical framework, selection of items and scales for the survey, and four studies that were conducted to revise the MDI and examine its psychometric properties. The findings indicate a theoretically predicted factor structure, high internal consistency, and document the convergent and discriminant validity of the MDI scales. The discussion delineates a plan for future validation studies that address further validity questions, such as predictive validity, measurement invariance, and fairness/bias, and provides a brief outlook of how the MDI may be used by practitioners, educators, and decision makers in schools and communities to motivate and inform action in support children’s well-being.

## Development and Validation of the Middle Years Development Instrument (MDI): Assessing Children’s Well-Being and Assets across Multiple Contexts

Over the past decades, there has been a burgeoning interest among researchers, educators, policymakers, and child-service agencies in monitoring children’s social and emotional development, health, and well-being at the population-level (e.g., Ben-Arieh 2008; Brown 2008; Keating and Hertzman 1999; Noll 2004). Such information can further our understanding of the conditions in which children grow up, and the individual and contextual mechanisms associated with well-being in childhood. Population-level indicators of children’s health and well-being can also inform programs, practices, and policies aimed at optimizing positive development and preventing educational and psychological difficulties (Land et al. 2011). Accordingly, there is a clear need for valid and reliable instruments that assess a range of domains of child well-being as well as the concomitant factors that underlie their promotion. To address this need, we created a population-level measure that (1) allows for an examination of associations between children’s social and emotional well-being and assets across multiple contexts, (2) is focused on the developmental period of “middle childhood,” (3) includes consideration of both positive and negative outcomes, and (4) gives children a voice in reporting on their own experiences and well-being.

Ben-Arieh (2005) argued that children should be involved “in measuring and monitoring their own well-being” (p. 574). Such an approach is aligned with the UN Convention on the Rights of the Child (www.unicef.org/crc/), Article 12, which states that “children are full-fledged persons who have the right to express their views in all matters affecting them and requires that those views be heard and given due weight in accordance with the child’s age and maturity.” Accordingly, one of our primary aims was to develop a reliable and valid instrument, using children as informants. The Middle Years Development Instrument (MDI) focuses on children’s social and emotional development, health, and well-being, and key contextual assets identified as important for developmental outcomes (e.g., Scales et al. 2006; Theokas and Lerner 2006). The MDI is designed to be administered as a large-scale, population-level measure so that stakeholders in communities and schools can obtain representative data on children during middle childhood on five dimensions: (1) Social and emotional development, (2) Connectedness to peers and adults at school, at home, and in the neighborhood, (3) School Experiences, (4) Physical health and well-being, and, (5) Constructive use of time after-school. In order to meet these criteria, the MDI team of researchers and community partners engaged in a 5-year development, piloting, and validation process, which is described in this paper.

The paper first presents (1) the lines of inquiry that informed the theoretical framework for the MDI; (2) our rationale for the focus on middle childhood; and (3) our previous research that set the stage for the MDI. Next, the paper describes the development of the MDI, including (1) a literature review and identification of developmentally important domains; (2) a review of survey and assessment tools in the domains of children’s social and emotional competence, physical and mental health and well-being, social relationships and connectedness with adults and peers, school experiences, and constructive use of after-school time; (3) a multi-stage consultation and piloting phase with children (grade 4), teachers and other educational staff, school administrators, parents/guardians, and youth program providers; and (4) the selection and refinement of items and response formats based on feedback from focus groups and on theoretical and psychometric criteria. Finally, the paper presents findings from psychometric validity analyses that are based on a school-district wide administration of the MDI.

### Theoretical Framework for the MDI

The theoretical framework underlying the MDI was derived from several literatures: social and emotional learning and development (Greenberg et al. 2003), positive psychology (Huebner et al. 2009; Seligman and Csikszentmihalyi 2000), resiliency and competence (Luthar 2006; Masten 2001; Masten and Coatsworth 1998), and a strengths- and asset-based approach to child development (Lerner et al. 2000; Theokas and Lerner 2006). Moreover, the development of the MDI was informed by ecological theories of human development (Bronfenbrenner 1979, 2005) and theories that emphasize the developmental primacy of social relationships (Ainsworth and Bowlby 1991; Ryan and Deci 2000; Thompson 1999). Both illustrate how social, biological, and cultural factors in different ecological contexts (e.g., family, school, community) jointly influence children’s development. Accordingly, the MDI was designed to obtain information about children’s relationships across multiple contexts (families, peers, adults in schools, neighborhoods), their experiences and activities in their primary social ecologies (family, school, community), and their social and emotional development, physical health, and well-being. Theoretical considerations that guided the selection of the constructs assessed with the MDI are provided in the literature review below.

### Importance of Middle Childhood

There are several reasons why middle childhood—between the ages of 6–12 years—is an especially important developmental period in which to study child well-being across multiple contexts both inside and outside of school. First, middle childhood, especially the ages between 10 and 12 years, is characterized by cognitive, social, emotional, and biological changes that set the stage for development in adolescence and adulthood (Eccles 1999). Although there is an abundance of research demonstrating that the early years are critical for healthy child development (e.g., Hertzman and Power 2006), it is during middle childhood that children’s personalities, behaviors, and competencies consolidate into forms that persist into adolescence and adulthood (Collins 1984). During this time, children master academic skills, such as reading, writing, and arithmetic, and also become more self aware, reflective, and planful. It is during middle childhood that children become less egocentric and are better able to consider the feelings and perspectives of others. At stake are the sense of right and wrong and the capacity to act in accordance with higher levels of social understanding. Moreover, children’s understanding of themselves and others becomes more complex during middle childhood. For instance, in early childhood children describe themselves in very concrete, observable characteristics (e.g., *I have brown hair*) and overt abilities or activities (e.g., *I am a good runner*). During middle childhood there is a move to describing the self more in terms of inner, psychological characteristics (e.g., *I am a person who gets angry easily*) and comparison to others (e.g., *I am the worst speller in my class*) (Harter 1990). The nature and pace of these changes make middle childhood an opportune time to identify modifiable factors associated with well-being and maladjustment so that appropriate prevention and interventions can be implemented to foster competence and deter the emergence of problems in adolescence.

Second, in middle childhood, children begin to spend more time in social settings outside of the family, such as schools and communities, that present them with new challenges that may influence their developmental pathways (Bianchi and Robinson 1997; Lancy and Grove 2011). Yet, there is a lack of research describing the ways in which the experiences that unfold across multiple ecological niches—including families, peer groups, schools, and neighborhoods—influence children’s social and emotional well-being.

Third, Erikson (1959) characterized middle childhood as a time of “industry versus inferiority” when the child’s attention is focused on acquiring new competencies and skills and learning how to get along with others, including peers and adults across a variety of contexts. In Erikson’s conceptualization of middle childhood, adults play a pivotal role in helping children develop a sense of “industry” (perceived usefulness and self-worth). If adults provide tasks that children perceive to be interesting, worthwhile, and accomplishable, children are more likely to develop a sense of their competency. Without opportunities to learn skills in supportive and caring contexts, however, they can develop a sense of “inferiority” (i.e., worthlessness, incompetence). Erikson also noted that it is during middle childhood that the radius of significant relations moves beyond the basic family to school and neighborhood social networks. Thus, how and with whom children spend their time in middle childhood may have important implications for opportunities and choices for time-use in later life when individuals have more autonomy to select developmental niches that can foster or deter their well-being. It is therefore important to understand middle childhood in the context of broader spheres of influence.

### The MDI Development: A Collaboration Based on Established Relationships

The development of the MDI grew out of several years of collaborations between university-based researchers, administrators and educational staff of the Vancouver school district, and members of a partnering community service organization, the United Way of the Lower Mainland. In these collaborations, community and school partners were involved in formulating research needs and questions, providing funding, supporting data collection, and disseminating research reports, as university researchers in turn gained experience in applying and communicating developmental and educational research to practitioners in locally meaningful and practice-relevant ways (Kershaw et al. 2005; Schonert-Reichl et al. 2010). Due to the established partnership, the MDI project members could draw from resources (time, information, funding, expertise, connections) in a way that none of the project members could have done by themselves. Most importantly, the partnership was characterized by mutual trust and positive interpersonal relationships, allowing team members to work through the challenges and intricacies that the large-scale partnership project entailed, and that are commonly encountered when researchers, practitioners, and (policy) decision makers collaborate (cf. Shonkoff 2000).

## Development of the MDI

### Identification of Developmentally Important Domains

An extensive literature review provided the basis for identifying the constructs and developmental domains that are, conceptually and empirically, considered to be essential in the development of competence and well-being in middle childhood and adolescence (e.g., Eccles 1999; Masten and Coatsworth 1998). In addition, educational staff and community program and service providers were consulted (via focus groups or surveys) to find out what self-report information from children would be particularly informative for their practices and programs.

Six broad domains were identified from this process: social and emotional development; physical health and well-being; connectedness (relationships with adults and peers); school experiences; after-school time use; and academic skills and achievement. The domain of academic skills and achievement was eventually not considered for inclusion in the MDI, because schools in British Columbia (BC) regularly collect students’ grades and data from standardized achievement tests (www.bced.gov.bc.ca/assessment/fsa/). Parenting and parent–child interactions are also critical factors in children’s development. However, these factors were not considered for inclusion in the MDI, because schools were unwilling to ask parenting questions in a survey administered in the public schools, as parents had expressed concern about their children being asked questions about private, personal family matters. In a second step, specific constructs were identified within each of the remaining five domains that would be included in the final instrument. Table 1 provides a list of constructs that were explored. The criteria for including or excluding constructs are described below.

**Table 1:** Domains and list of constructs/scales reviewed and considered for the MDI

Domain	Constructs
Social and emotional development	Empathy, optimism, general self-concept, psychological well-being (anxiety/worries; depression/sadness), satisfaction with life, prosocial behavior, *trust, resiliency, perspective taking, social competence, self*-*efficacy, honesty, emotion regulation, social responsibility, altruism*
Connectedness (relationships with adults and peers)	Sense of support and belonging with adults at home, at school, and in the neighborhood/community, number of important adults at school, *characteristics of the important adults in school,* availability of safe places in the community for children, availability of programs for children, peer belonging, friendship intimacy
School experiences	Academic self-efficacy, school climate/supportiveness, school belonging, future goals and ambitions, motivation in school, victimization (bullying), *school self*-*concept, classroom autonomy, school liking, personal power, parental help, parent support*
Physical health and well-being	Overall health, physical health conditions, body image, health habits (nutrition, sleep, *dental hygiene), perceived pressure (stress), somatization, physical activity*
Use of after-school time	After school time (what, where, with whom): participation in organized activities (education, arts & music, volunteering/youth clubs, individual sports, team sports), other activities (sports, homework, TV/videos, computer games, instant messaging/emails, reading, chores, music practice, arts & crafts, hang out with friends), desired after-school activities, barriers to after-school time activities

Constructs in regular font were included in the MDI; constructs in italics were considered for inclusion but for various reasons (as described in the text) were not included

### Selection of Items and Scales

The next step was to identify reliable and valid items and/or scales for the constructs identified in Table 1. The criteria for consideration were (1) high reliability (i.e., Cronbach’s alpha ≥.7) in previous research, (2) evidence of discriminant, convergent and content validity from existing data, and (3) age-appropriateness (age 9–12) of item content and wording. The original list of candidates included over 300 items, from more than 100 scales, including scales that MDI researchers had used in previous research (Schonert-Reichl et al. 2007), as well as scales that had been used in other large-scale projects on middle childhood and adolescence. Scales that did not meet the above criteria were excluded. Also, when two or more scales assessed similar constructs and were highly correlated, the scale with the best psychometric properties and broadest validity evidence was retained. For scales that consisted of more than three items, results of previously conducted factor analyses and reliability assessments (i.e., factor loadings, Cronbach’s alpha after an item is deleted) were used to guide data reduction, in order to shorten scales to a maximum of three to five items.

Throughout this process, decisions were made in consultation with all MDI project partners, their broad range of expertise in psychometric research, assessment, questionnaire development, developmental theory, and child development. Our school and community partners provided insights regarding which items and scales would be considered face valid and of particular interest to or lead to potential conflict with parents, teachers, school administrators, policy makers, and/or ministry of education delegates. The resulting selection of items/scales was circulated among a group of parents, educational staff, and school board administrators, who were not directly involved in the development of the MDI. Based on their feedback, a few additional revisions were made (e.g., rewording instructions; adding definitions of the words *bullying* and *community/neighborhood*).

## Four Studies to Validate and Revise the MDI

Previous research has found that surveys can be administered with reliability and validity to 9- to 10-year old children in the areas of social and emotional development, school experiences, relatedness (e.g., Battistich et al. 1997; Developmental Studies Center 2001), health (e.g., WestEd 2011), and after-school time use (Schonert-Reichl et al. 2007). There are, however, few studies that jointly survey all (or several) of these domains at a representative population level. Exceptions include the *World Vision Kinderstudie*, conducted in Germany by Hurrelmann and Andresen in 2007, and the Search Institute’s work on Developmental Assets in the United States (e.g., Scales et al. 2006).

For the purpose of revising and validating our newly developed survey, four studies were conducted: Study 1: Administration of initial version of the MDI, conducted by trained research assistants, and follow-up focus groups with children upon their completion of the survey. Study 2: Administration of revised MDI, conducted by teachers, and follow-up focus groups with teachers. Study 3: District-wide implementation of the MDI in one school district. Study 4: District-wide implementation of the MDI in two other school districts. The following sections describe the key methods and findings of each study, including the MDI revisions made. To avoid redundancy, rather than providing details on items and scales for each study, the final version of the MDI, including all items/scales, is described as part of Study 4.

### Study 1: Administration of Initial MDI Version and Child Focus Groups

The first version of the MDI included 96 items, plus eight demographic questions, and was administered (i.e., read out loud in class) in March 2008 by research assistants to five grade 4 and 5 classrooms in five schools (N = 108). The data obtained were examined with regard to (1) missing data (e.g., high degrees of missing data on items may suggest that an item is difficult, unclear, or incomprehensible); (2) response patterns (e.g., checking only the highest or lowest response options may reflect a lack of understanding or interest); (3) skewness and distributions of response options (to examine whether children used the entire response scale and/or to see whether items discriminated among students); and (4) item-level factor loadings and scale reliabilities (Cronbach’s alpha and ordinal alpha [cf. Zumbo et al. 2007; Gadermann et al. 2012]). In addition, two research assistants conducted 17 focus groups, with two research assistants and four to six children in each group. Children were asked to provide feedback on the length of the survey, the understandability of the instructions, individual items, response options, and on the content. Also, children were encouraged to comment and provide suggestions on anything that they noticed during the MDI administration.

Based on the psychometric analyses and the feedback received from students, the MDI was shortened to 71 items, and some changes were made to items, formatting, and wording (e.g., reformatting time-use questions; simplifying response scale formats; re-wording definitions). Overall, the feedback from the students was positive. Children enjoyed taking the survey and felt that it was important to be asked these questions, as reflected in comments like, “It was good, you can write on paper on how you feel without having to talk about it in person, and because it is in secret” or “I liked it because we could share what we thought”. A few students voiced concern that some items (e.g., on being bullied or body weight) may make “children […] feel bad when they are asked these questions”. However, after the research assistants explained the purposes of the survey—to implement initiatives to support children—the children expressed that having the opportunity to report confidentially about uncomfortable issues would be better than keeping silent. Based on this feedback, a statement regarding the survey responses’ confidentiality and the following sentence was inserted at the beginning of the survey: “Your answers are very important and will help improve programs for children your age.”

### Study 2: Teacher Administration of the MDI and Teacher Focus Groups

In June 2008, a revised version of the MDI, containing eight demographic background items and 71 survey items, was read out loud in class, by classroom teachers to 80 fourth graders from seven classrooms in three elementary schools. Teachers received a teacher manual describing how to administer the MDI prior to data collection. Following administration, five volunteering teachers were interviewed in two focus groups to provide feedback on the MDI survey, the administration, and the manual. Based on their feedback, the teacher manual was simplified and streamlined to make it more user-friendly. Teacher feedback regarding the MDI was very positive. They reported that students seemed to enjoy the MDI, and that it “hits a really important topic with kids of this age”. One teacher described the MDI as “an important tool to help teachers better understand students and as a tool to talk to communities about specific needs and possible ways that help is needed”. Preliminary analyses of the factor structure and the reliabilities of scales indicated good psychometric properties of the revised MDI scales. Given the small sample size, however, no empirical details are reported. Details on the psychometric findings are presented as part of Studies 3 and 4.

### Study 3: District-Wide Administration of the MDI

In January 2010, the MDI was administered in a large urban public school district in BC, Canada. Prior to administration, letters of approval were obtained from the district’s School Board, the BC Ministry of Education, the district’s Elementary School Teachers Association, and the ethics board of the University of BC, and the application for research ethics approval contained detailed references to the BC’s Freedom of Information and Protection of Privacy Act and Personal Information Protection Act (see www.oipc.bc.ca). These references were legally required, because the MDI was administered with passive parental consent (see details below), and because the MDI collects information that identifies individual children (i.e., the personal education number and the postal code of children’s home residence). (For data analysis, all data were converted into anonymized files.)

#### Sample

Participants included 3,026 grade 4 students (48 % girls; *mean age* = 9.7, *SD* = .3), from 201 classrooms in 72 (out of 81) elementary schools of a diverse, urban public school district (with a total student population of over 50,000), representing 80 % of the district’s total grade 4 population. English was reported as the first language learned by 40 % of the participants, with 33 % reporting that they first learned a language other than English, and 27 % reporting one or more “first languages”. Participation was voluntary for each school and teacher. Passive parental consent procedures were used; parents/guardians were informed about the MDI prior to its administration via letters, and they could withdraw their children from the study by sending a note to the school. Children’s participation was voluntary, and children were asked for their assent. Student participation rate was 93 % overall. All participating teachers (n = 201) were invited to provide feedback via telephone interviews and/or surveys following the MDI administration. Participation was voluntary, and the response rate was 24 % (n = 48).

#### Procedure

The MDI survey was administered by classroom teachers, who received an administration manual. The teachers read aloud a prepared statement regarding student assent, informing students that participation was voluntary and responses confidential, and that there were no consequences for not participating. To reduce biases due to children’s variable reading proficiencies, the teacher read each item aloud, and students were encouraged to ask questions as needed. On average, children completed the MDI in two 40-minute class periods. Afterwards, written feedback regarding the MDI and its administration was solicited from teachers.

#### Measures

The MDI contains seven demographic questions (gender, birth date, adult(s) the child lives with, number of siblings, first language(s) learned, language(s) spoken at home, English reading proficiency) and 71 items that assess five domains of children’s development and well-being: (1) Social and emotional development, (2) Connectedness to peers and to adults at school, at home, and in the neighborhood, (3) School experiences, (4) Physical health and well-being, and (5) Constructive use of time after school. Each domain is comprised of several subscales and/or individual items. Because the questionnaire asks questions about peer relationships, bullying/victimization, and school supportiveness, children may indicate at the end of the questionnaire, on a separate, detachable sheet, whether they “want help with problems [they] are having with other students”. Table 2 presents all items/scales, response scale formats, and references to the original sources for the items and scales. (We note that all items in Table 2 were used in Studies 3 and 4; however, as described below, some response formats and the order of scales were changed between Studies 3 and 4. Table 2 contains the final version of all items.)

**Table 2:** MDI items/scales, scale mean scores, alphas, response formats, CFA factor loadings, item means, standard deviations (all based on data from Study 4) and original sources and references of items and scales

**Domain**	
*Construct* ( *scale mean, Cronbach’s α*, *ordinal α*)	Response format
Item wording	CFA factor loading (item mean; standard deviation)
	*Original source of item(s)/scale(s)* (reference(s))
**Social and emotional development**	
*Empathy* ( *M* = 4.3; *SD* = .7; *α* = .65; *ordinal α* = .73)	‘Disagree a lot’ = 1 to ‘Agree a lot’ = 5
1. I feel sorry for other kids who don’t have the things that I have	.60 (*M* = 4.2; *SD* = 1.0)
2. When I see someone being treated mean it bothers me	.65 (*M* = 4.3; *SD* = 1.0)
3. I am a person who cares about the feelings of others	.83 (*M* = 4.4; *SD* = .9)
	*Interpersonal Reactivity Index* (Davis 1983; modified by Eisenberg et al. 2002)
*Optimism* ( *M* = 4.0; *SD* = .8; *α* = .66; *ordinal α* = .70)	‘Disagree a lot’ = 1 to ‘Agree a lot’ = 5
4. I have more good times than bad times	.72 (*M* = 4.0; *SD* = 1.1)
5. I believe more good things than bad things will happen to me	.68 (*M* = 4.1; *SD* = 1.1)
6. I start most days thinking I will have a good day	.59 (*M* = 4.1; *SD* = 1.1)
	*Resiliency Inventory Subscale* (Noam and Goldstein 1998; Oberle et al. 2010; Song 2003)
*General self* - *concept* ( *M* = 4.4; *SD* = .7; *α* = .72; *ordinal α* = .79)	‘Disagree a lot’ = 1 to ‘Agree a lot’ = 5
7. In general, I like being the way I am	.76 (*M* = 4.5; *SD* = .9)
8. Overall, I have a lot to be proud of	.73 (*M* = 4.4; *SD* = .9)
9. A lot of things about me are good	.75 (*M* = 4.4; *SD* = .9)
	*Self Description Questionnaire (SDQ)*, (Marsh 1988)
*Sadness (Depr. symptoms)* ( *M* = 2.4; *SD* = 1.0; *α* = .70; *ordinal α* = .75)	‘Disagree a lot’ = 1 to ‘Agree a lot’ = 5
10. I feel unhappy a lot of the time	.78 (*M* = 2.2; *SD* = 1.2)
11. I feel upset about things	.63 (*M* = 2.6; *SD* = 1.2)
12. I feel that I do things wrong a lot	.71 (*M* = 2.4; *SD* = 1.3)
*Worries (Anxiety symptoms)* ( *M* = 2.9; *SD* = 1.2; *α* = .80; *ordinal α* = .85)	
13. I worry about what other kids might be saying about me	.81 (*M* = 3.2; *SD* = 1.5)
14. I worry a lot that other people might not like me	.86 (*M* = 2.7; *SD* = 1.4)
15. I worry about being teased	.76 (*M* = 2.7; *SD* = 1.5)
	*Seattle Personality Questionnaire* (Kusche et al. 1988; Rains 2003)
*Satisfaction with life* ( *M* = 4.1; *SD* = *.9; α* = .82; *ordinal α* = .88)	‘Disagree a lot’ = 1 to ‘Agree a lot’ = 5
16. In most ways my life is close to the way I would want it to be	.71 (*M* = 3.9; *SD* = 1.2)
17. The things in my life are excellent	.85 (*M* = 4.1; *SD* = 1.1)
18. I am happy with my life	.90 (*M* = 4.4; *SD* = 1.0)
19. So far I have gotten the important things I want in life	.68 (*M* = 4.2; *SD* = 1.0)
20. If I could live my life over, I would have it the same way	.69 (*M* = 3.7; *SD* = 1.4)
	Modified from *Satisfaction with Life Scale* (Diener et al. 1985; Gadermann et al. 2010)
*Prosocial behavior* ( *M* = 3.0; *SD* = 1.1; *α* = .82; *ordinal α* = .85)	‘Never’ = 1 to ‘Many times’ = 1
Since the start of this school year, …	
21. … I cheered someone up who was feeling sad	.83 (*M* = 3.1; *SD* = 1.2)
22. … I helped someone who was being picked on	.76 (*M* = 2.7; *SD* = 1.3)
23. … I helped someone who was hurt	.84 (*M* = 3.2; *SD* = 1.2)
	*Youth Outcome Measures for AfterSchool KidzLit™* (Developmental Studies Center 2001)
**Connectedness**	
*Important adults in my school*	‘Yes’ = 1 and ‘No’ = 0
24. Are there any adults who are important to you at your school?	na (*M* = .80; *SD* = .4)
(If ‘yes’, list all the adults who are important to you at your school.)	na (*M* = 3.2; *SD* = 1.2)
	(Blyth et al. 1982; modified by Schonert-Reichl and Buote 2004)
*Adults at school* ( *M* = 3.2; *SD* = .7; *α* = .71; *ordinal α* = .77)	‘Not at all true’ = 1 to ‘Very much true’ = 4
At my school, there is a teacher or another adult …	
25. … who really cares about me	.76 (*M* = 3.1; *SD* = .9)
26. … who believes that I will be a success	.77 (*M* = 3.2; *SD* = .9)
27. … who listens to me when I have something to say	.70 (*M* = 3.4; *SD* = .8)
*Adults at home* ( *M* = 3.6; *SD* = .5; *α* = .69; *ordinal α* = .80)	
In my home, there is a parent or another adult…	
28. … who believes that I will be a success	.89 (*M* = 3.7; *SD* = .6)
29. … who listens to me when I have something to say	.92 (*M* = 3.6; *SD* = .7)
30. … who I can talk to about my problems	.83 (*M* = 3.6; *SD* = .8)
*Adults in neighborhood/community* ( *M* = 2.8; *SD* = 1.0; *α* = .87; *ordinal α* = .91)	
In my neighborhood (not from your school or family), there is an adult…	
32. … who really cares about me	.79 (*M* = 2.9; *SD* = 1.1)
33. … who believes that I will be a success	.78 (*M* = 2.8; *SD* = 1.1)
34. … who listens to me when I have something to say	.72 (*M* = 2.9; *SD* = 1.1)
	*California Healthy Kids Survey* (Constantine and Benard 2001; Hanson and Kim 2007; WestEd 2011).
*Relationships with parents/guardians*	‘Never’ = 1 to ‘Always’ = 4
31. I care about what my parents (or guardians) think of me	na (*M* = 3.6; *SD* = .7)
	Modified from *Health Behavior in School*-*Aged Children (HBSC),* (Health Canada 1999)
*My neighborhood community*	
35. Are there places in your neighborhood that provide programs for kids your age, like sports (for example, …)?	No (6 %); Yes (74 %); Don’t know (20 %)
36. Are there safe places in your neighborhood where you feel comfortable to hang out with friends, like …?	No (6 %); Yes (77 %); Don’t know (17 %)
	*Chapin Hall*, University of Chicago (Goerge and Chaskin 2004)
*Peer belonging* ( *M* = 4.1; *SD* = .9; *α* = .79; *ordinal α* = .84)	‘Disagree a lot’ = 1 to ‘Agree a lot’ = 5
37. I feel part of a group of friends that do things together	.77 (*M* = 4.2; *SD* = 1.1)
38. I feel that I usually fit in with other kids around me	.82 (*M* = 4.0; *SD* = 1.1)
39. When I am with other kids my age, I feel I belong	.81 (*M* = 4.2; *SD* = 1.1)
*Friendship intimacy* ( *M* = 4.3; *SD* = 1.0; *α* = .79; *ordinal α* = .86)	‘Disagree a lot’ = 1 to ‘Agree a lot’ = 5
40. I have at least one really good friend I can talk to when something is bothering me	.87 (*M* = 4.5; *SD* = 1.1)
41. I have a friend I can tell everything to	.80 (*M* = 4.1; *SD* = 1.3)
42. There is somebody my age who really understands me	.83 (*M* = 4.2; *SD* = 1.2)
	*Relational Provisional Loneliness Questionnaire (RPLQ)* (Hayden-Thomson 1989)
**School experiences**	
*Acad. Self* - *Efficacy* ( *M* = 4.4; *SD* = .7; *α* = *.79*; *ordinal α* = .82)	‘Disagree a lot’ = 1 to ‘Agree a lot’ = 5
43. I am certain I can learn the skills taught in school this year	.79 (*M* = 4.3; *SD* = .9)
44. If I have enough time, I can do a good job on all my school work	.70 (*M* = 4.5; *SD* = .9)
45. Even if the work in school is hard, I can learn it	.79 (*M* = 4.3; *SD* = .9)
	*Self Beliefs/Academic Self*-*Efficacy* (Roeser et al. 1996)
*Supp. school environm.* ( *M* = 4.1; *SD* = .9; *α* = .74; *ord. α* = .80)	‘Disagree a lot’ = 1 to ‘Agree a lot’ = 5
46. Teachers and students treat each other with respect in this school	.78 (*M* = 4.3; *SD* = 1.0)
47. People care about each other in this school	.84 (*M* = 4.2; *SD* = 1.0)
48. Students in this school help each other, even if they are not friends	.68 (*M* = 3.9; *SD* = 1.2)
	*School Climate* (Battistich et al. 1997)
*School belonging*	‘Disagree a lot’ = 1 to ‘Agree a lot’ = 5
49. I feel like I belong in this school	na (*M* = 4.3; *SD* = 1.1)
50. I feel like I am important to this school	na (*M* = 3.8; *SD* = 1.2)
	*Relatedness/School Belonging* (Roeser et al. 1996)
Future Goals & Ambitions	‘Disagree a lot’ = 1 to ‘Agree a lot’ = 5
51. When I grow up, I have goals and plans for the future	na (*M* = 4.5; *SD* = 1.0)
	*Resilience & Youth Development Module,* *California Healthy Kids Survey* (Hanson and Kim 2007; WestEd 2011)
Motivation	‘Not at all true of me’ = 1 to ‘Very true of me’ = 4
How important is it to you to do the following in school:	
52a. Make friends?	na (*M* = 3.7; *SD* = .6)
52b. Get good grades?	na (*M* = 3.8; *SD* = .5)
52c. Learn new things?	na (*M* = 3.7; *SD* = .6)
	*National Longitudinal Study of Children and Youth* (Statistics Canada 1997)
Victimization ( *M* = 1.7; *SD* = .8; *α* = .75; ordinal *α* = .81)	‘Not at all’ = 1 to ‘Several times a week’ = 5
53. Physical Bullying	.75 (*M* = 1.7; *SD* = .9)
54. Verbal Bullying	.83 (*M* = 1.9; *SD* = 1.1)
55. Social Bullying	.78 (*M* = 1.9; *SD* = 1.1)
56. Cyberbullying	.55 (*M* = 1.2; *SD* = .7)
	Modified from *Safe School Student Survey*, Grades 4–7, (Law et al. 2011; Trach et al. 2010)
**Physical health and well-being**	
*Overall health & health condition*	‘Poor’ = 1 to ‘Excellent’ = 4
57. In general, how would you describe your health?	na (*M* = 3.5; *SD* = .6)
58. Do you have a physical or health condition that keeps you from doing some things other kids your age do?	Yes (13 %) and No (87 %)
	*Youth Health Survey* (McCreary Centre Society 2009)
*Body image*	‘Very underweight’ = 1 to ‘very overweight’ = 5
59. How do you rate your body weight?	na (*M* = 2.9; *SD* = .7)
	*California Healthy Kids Survey* (Constantine and Benard 2001; Hanson and Kim 2007; WestEd 2011)
	‘Never’ = 1 to ‘always’ = 5
60. How often do you like the way you look?	na (*M* = 4.0; *SD* = 1.0)
	*National Longitudinal Study of Children and Youth* (Statistics Canada 1997)
*Nutrition & sleep*	‘Never’ = 0, ‘1 day per week’, …, to ‘Every day’ = 7)
61. How often do you eat breakfast?	na (*M* = 6.5; *SD* = 1.5)
62. How often do your parents or another adult family member eat meals with you?	na (*M* = 5; *SD* = 2.1)
63. How often do you eat food like pop, candy, potato chips, or something else?	na (*M* = 2.8; *SD* = 2.1)
64. How often do you get a good night’s sleep?	na (*M* = 5.2; *SD* = 2.1)
	‘Before 9:00 pm’ = 1 to ‘After midnight’ = 5
65. What time do you usually go to bed during the weekdays?	na (*M* = 2.0; *SD* = 1.0)
	*United Way of the Lower Mainland Daily Diary* (Schonert-Reichl 2007)
**Constructive use of time**	
*With whom and where*	
66. On school days, who are you usually with for most of the afternoon (from after school to 6 pm)?	By myself (13 %); friend(s) (29 %); mother (66 %); father (42 %); younger siblings (27 %); older siblings (31 %); other adult(s) (18 %); other (14 %)
	‘Never’ = 0 to ‘5 times a week’ = 5
How many days a week do you go to these places after school (3:00 to 6:00 pm)?	
67a. I go home	na (*M* = 4.8; *SD* = 1.6)
67b. I stay at school to participate in afterschool activities	na (*M* = 1.8; *SD* = 1.3)
67c. I go to an afterschool program/daycare	na (*M* = 2.2; *SD* = 1.7)
67d. I go to a friend’s house	na (*M* = 2.2; *SD* = 1.2)
67e. I go to a park, playground, or community centre	na (*M* = 2.0; *SD* = 1.3)
67f. I hang out at the mall or stores	na (*M* = 1.6; *SD* = 1.0)
67 g. I go someplace else ([…])	na (*M* = 1.9; *SD* = 1.3)
*Organized activities*	
During last week after school (3:00 to 6:00 pm), did you participate in:	‘Never’ = 0 to ‘5 times a week’ = 5
68a. Educational lessons or activities, such as…?	na (*M* = 1.7; *SD* = 1.2)
68b. Art or music lessons, such as…?	na (*M* = 1.6; *SD* = 1.0)
68c. Youth organizations, such as…?	na (*M* = 1.2; *SD* = .7)
68d. Individual sports with a coach or instructor, such as…?	na (*M* = 2.0; *SD* = 1.4)
68e. Teams sports with a coach or instructor, such as…?	na (*M* = 2.2; *SD* = 1.5)
*Other after school activities*	‘Never’ = 0 to ‘5 times a week’ = 5 & ‘Less than 30 min’ = 1 to ‘2 or more hrs’ = 4
69. (a) During last week after school (3:00 to 6:00 pm), did you … (i) do sports and/or exercise for fun?; (ii) do homework? (iii) watch TV? (iv) play video or computer games?; (v) instant Message?; (vi) read for fun?; (vii) do household chores?; (viii) practice a musical instrument?; (ix) do Arts & Crafts?; (x) hang out with friends?	*[Data available on request]*
(b) About how much time did you usually spend doing—the activity on one of those days?	
*Desired after* - *school activities*	
70. Think about what you want to do after school from 3 pm to 6 pm. Are you already doing the activity you want to be doing?	Yes (55 %); No (27 %); Yes and No (18 %)
If no, list one of activity you wish you could do	*[Data available on request]*
Where would you like this activity to be?	*[Data available on request]*
School; Home; Park or playground; Community centre; Other	
If yes, list one activity that you are already doing (and want to be doing)?	*[Data available on request]*
Where do you do this activity?	*[Data available on request]*
School; Home; Park or playground; Community centre; Other	
*Barriers*	Checklist (check all that apply)
71. What stops you from participating in the activities that you want to participate in after school?	
(a) I have to go straight home after school; (b) It is too difficult to get there; (c) The activity that I want is not offered; (d) The schedule does not fit the times that I can attend; (e) It’s not safe for me to go; (f) I have too much homework to do; (g) My parents do not approve; (h) It costs too much; (i) I need to take care of brothers or sisters or do other things at home; (j) I am afraid I will not be good enough in that activity; (k) I’m too busy; (l) I don’t know what is available; (m) None of my friends are interested or want to go; (n) Other, please describe __________	*[Data available on request]*
	*Chapin Hall*, University of Chicago, (Goerge and Chaskin 2004)

##### *School*- *and Neighborhood*-*level Aggregation and Data Linkage*^1^

For every child completing the MDI, information from administrative school records was obtained: school name/ID, district name/ID, children’s residential 6-digit postal code, and children’s Personal Education Number. The information is used to provide every participating school/district with a report on the aggregated results, comparing school/district results with the district’s/province’s average. The 6-digit postal code was used to aggregate MDI according to children’s residential neighborhoods, and to link MDI data to neighborhood-level data on socio-economic and demographic context characteristics. Children’s Personal Education Numbers were obtained to link MDI data to other available educational data (e.g., teacher reports on developmental outcomes in kindergarten; academic grades). The MDI project thus allows us for the creation of databases with which developmental trajectories can be explored.

#### Analyses

The factor structure of the MDI was evaluated using exploratory factor analysis (EFA) for the three domains of the MDI that contain scales.^2^ (1) social and emotional development, (2) connectedness, and (3) school experiences. Items in each domain were factor analyzed separately. To determine the number of factors, parallel analysis and interpretability of factors were used. Parallel analyses were conducted using the procedure developed by Presaghi and Desimoni (2011) in *R*, which accommodates ordinal and categorical data (i.e., Likert-type response formats) by utilizing the polychoric correlation matrices. In all analyses, the number of replications for the random data simulations was set to 5, and the quantile to .99. The EFAs were performed in MPlus (version 6, Muthén and Muthén 2010) on the polychoric correlation matrices to accommodate the Likert-type data. All analyses used the mean and variance adjusted weighted-least squares estimation method (WLSMV). Factors were rotated using oblique (geomin) factor rotation.

### Results (Study 3)

Results from the parallel analyses and EFAs for each of the three examined MDI domains are presented in turn, followed by a summary of teacher feedback received regarding administration of the MDI.

#### *Social and Emotional Development*

The social and emotional development domain is assessed using 23 items, with three items each for six constructs (empathy, optimism, prosocial behavior, self-concept, sadness, and anxiety), and five items from the Satisfaction with Life Scale for Children. The parallel analysis identified seven factors and the EFA showed simple structure for all seven original constructs except self-concept. The three items of the self-concept scale had loadings of .20, .35, and .63 on one factor, and loadings of .42, .37, and .27 on the Satisfaction with Life Scale factor. (We note that the loadings and cross-loadings for the self-concept scale are considered low—please see Study 4 for further comments.) The factors for satisfaction with life, optimism, and self-concept correlated highly (r = .48 to .64), as did the factors for sadness and anxiety (r = .55). The seven factors explained 69 % of the total variance.

#### *Connectedness*

The connectedness domain contains 19 items. Of these, four items are single-item measures (e.g., safe places in the neighborhood; school belonging), and three items each pertain to five scales: connectedness with adults at (1) home, (2) in school, and (3) in the neighborhood, (4) peer belonging, and (5) friendship intimacy. The latter 15 items were used in the EFA. The parallel analysis with the 15 items suggested six factors for extraction. However, this would indicate that one or more factors would be under-identified (i.e., have less than three items loading primarily on that factor). In fact, when running a six-factor solution, the sixth factor could not be interpreted, with only one item loading on it. Accordingly, the analysis was re-run with 5 factors, and all items loaded highly on their primary factors. The total variance explained was 77 %. Correlations among factors ranged from .33 to .55.

#### *School Experiences*

The school experiences domain contains 14 items. Of these, four items are single-item measures (e.g., future goals), so that only 10 items were included in the EFA, with three items each for (1) school supportiveness, and (2) academic self-efficacy, and four items for victimization. The parallel analysis identified three factors as the optimal solution, explaining 72 % of the total variance, and the EFA showed simple structure, with high primary loadings, and low cross-loadings. In this domain, victimization correlated −.31 with school supportiveness and −.10 with academic self-efficacy, and the latter two correlated .47.

#### *Teacher Feedback*

The overall feedback from teachers was very positive. Several teachers recognized that the MDI gives students a voice, because it is “a chance for students to express an opinion” and gives them “the feeling that their opinion matters”. Also, teachers stated that it allowed students time to reflect and “identify what may be troubling them”. Teachers also stated that the MDI provides useful and important information about students and communities with the potential to encourage changes that will benefit students. Furthermore, teachers stated that the domains addressed in the MDI are consistent with the school districts’ mandate to foster ‘social responsibility’ in their classrooms.

Teacher feedback also verified the feasibility of administering the MDI to grade 4 students, although several teachers raised concerns about the length of administration. Accordingly, teachers suggested making response formats consistent across scales, to reduce the time needed to redirect student attention to changes in response options. Teachers also suggested visually and verbally simplifying response options for the time use section. As described below, teacher feedback and empirical findings were used to make final changes to the MDI.

### Study 4: Scaling up—District-Wide Implementation in Two School Districts

In February 2011, the MDI was implemented in two different school districts in BC, providing an opportunity to test whether the population-level MDI data collection and dissemination procedures would be functional in, and attractive to, school districts that had not been partners in the development of the MDI. Also, findings from this study allowed us to examine the factor structure and psychometric properties of the final version of the MDI.

### Method (Study 4)

#### Sample

Participants included 2,000 grade 4 students (49 % girls; *mean age* = 9.8, *SD* = .3) from all 45 public elementary schools of a diverse, (sub)urban public school district (n = 1,921) and all five elementary schools of a small rural school district (n = 79) in BC, Canada, with student participation rates of 88, and 99 %, respectively. The majority of students (64 %) identified English as their only first language, 20 % reported a language other than English, and 16 % reported first learning English and one (or more) other language(s), including .3 % who reported an Aboriginal language as their first language.

#### Procedure

The procedures for parental consent, student assent, and MDI administration by teachers were the same as in Study 3.

#### Measures

The MDI survey remained identical except for changes to (1) response formats of several scales, (2) sequence/order of items, (3) graphical design, and (4) first language learned and language spoken at home questions. According to the feedback obtained in Study 3, the response format of 10 scales were changed to ‘disagree a lot’, disagree a little’, ‘don’t agree or disagree’, agree a little’, and ‘agree a lot’. Also, given the high cross-loadings of items from the self-concept and satisfaction with life scales, the order of presentation was changed, so that these two scales did not follow one another. Also, the time use section was graphically revised, with the response format changed from ‘no’/’yes’ and (if yes) ‘1–2 days’, ‘3–4 days per week’, etc., to ‘0/never’, ‘1 day’, ‘2 days per week’, etc. Also, one response option (‘Once or a few times’) was added to the victimization scale (‘Not at all this school year’, ‘About every month’; ‘About every week’, and ‘Many times a week’), given evidence from Study 3 of non-normal distributions for these response options. Finally, the language background questions—(1) ‘What is the first language you learned at home?’ and (2) ‘Which language(s) do you speak at home?’—were expanded so children could check the option ‘Aboriginal language’ and write down their Aboriginal language, if applicable. (This addition reflected recommendations by an Aboriginal Steering Committee that consults our research group regarding projects involving children from Aboriginal backgrounds.)

#### Analyses

First, confirmatory factor analyses (CFAs) were conducted in MPlus, to test the model fit of the factor solutions indicated in Study 3, using polychoric correlation matrices, the WLSMV estimation method, and the oblique (geomin) factor rotation. Model fit was evaluated using the following criteria: (1) a root mean square error of approximation (RMSEA) <.06 (Beauducel and Herzberg 2006), (2) a comparative fit index (CFI) ≥.95 (Yu 2002), and (3) a Tucker-Lewis Index (TLI) of ≥.95 (Hu and Bentler 1999; Schumacker and Lomax 2004; Vandenberg and Lance 2000).

Second, correlations among subscale scores were computed within each of the three factor-analyzed MDI domains,^3^ and then the online tool on the *SISA* website (Uitenbroek 1997) was used to examine whether convergent correlations were significantly higher than discriminant correlations.

### Results (Study 4)

#### *Confirmatory Factor Analyses*

The results from the CFAs all indicated good model fit. For the social and emotional development domain, the CFI was .96, the TLI .98, the RMSEA .05, and factor loadings ranged from .59 to .90. For the connectedness domain, the CFI was .98, the TLI .99, and the RMSEA .05, with factor loadings ranging from .70 to .92. For the school experiences domain, the CFI was .99, the TLI .99, and the RMSEA was .04, with factor loadings from .68 to .84, with the exception of a .55 loading for the item on cyber-bullying. Factor loadings at .4 are commonly considered a lower bound for including items in a factor (Ford et al. 1986). Factor loadings (where applicable), internal consistency (Cronbach’s alpha, ordinal alpha), as well as the scale and item means and standard deviations are presented in Table 2.

#### *Convergent and Discriminant Validity*

Table 3 shows the correlations between scales within the Social and emotional development domain, the Connectedness domain, and the School experiences domain, respectively. Correlations with an absolute value between .24 and .36 are bolded, to indicate that they reflect a medium effect size, and correlations greater than .37 are bolded and underlined, reflecting a large effect size (Becker 2000).

**Table 3:** Pearson correlations of scales within the social and emotional development domain, within the connectedness domain, and within the school experiences domain

Pearson correlations of scale mean scores	1	2	3	4	5	6
Social and emotional development domain	1	2	3	4	5	6
Empathy						
Prosocial behavior	**.36**					
Optimism	**.28**	.21				
Self concept	**.34**	.23	**.58**			
Life satisfaction	.23	.18	**.57**	**.62**		
Sadness (depression)	−.06	−.05	− **.40**	−**.34**	**−.40**	
Worries (anxiety)	.05	.08	−.20	−.19	−**.27**	**.48**
Connectedness domain	1	2	3	4		
Adults at school						
Adults in neighborhood	**.40**					
Adults at home	**.38**	**.27**				
Peer belonging	**.35**	**.29**	**.33**			
Friendship intimacy	**.30**	**.29**	**.30**	**.48**		
School experiences domain	1	2	3	4		
Academic self-efficacy	**.38**					
School supportiveness	**.39**	**.40**				
School belonging	**.42**	**.46**	**.59**			
Bullying/victimization	−.06	−.09	−.23	−**.24**		

All correlations are statistically significant at the .01 level. Correlations equivalent to a (1) medium effect size are bolded and (2) large effect size bolded and underlined

#### *Social and Emotional Development*

The correlation between the theoretically-related scales of ‘empathy’ and ‘prosocial behavior’ (*r* = .36) was significantly^4^ higher than these scales’ correlations with any other construct in the Social and emotional development domain, except for the correlation between empathy and self-concept (r = .34). The correlations between the conceptually and empirically related scales for ‘optimism’, ‘self-concept’, and ‘satisfaction with life’ were large (.57 to .62), and significantly higher than the correlations with all other constructs (−40 to .34; cf. Gadermann et al. 2010). Likewise, the two subscales ‘sadness’ (depressive symptoms) and ‘worries’ (anxiety symptoms) correlated significantly higher with each other (*r* = .48) than with all other constructs in the domain (−.40 to .08).

#### *Connectedness*

The two peer relationship scales (‘peer belonging’ and ‘friendship intimacy’) correlated significantly more highly with each other (r = .48) than with any adult relationship scales (.29 to .35). Also, the correlation of ‘adults in the neighborhood’ with ‘adults at school’ (r = .40) was significantly higher than any correlation between the peer and adult relationship scales (.29 to .35). The correlation between ‘adults at home’ and ‘adults at school’ (r = .38) was higher than all correlations between the peer and adult relationship scales (.29 to .35), but the differences were not consistently statistically significant.

#### *School Experiences*

The scales for ‘academic self-efficacy’, ‘school belonging’, and ‘school supportiveness’ were all highly correlated with on another (.39 to .59), and negatively correlated with ‘bullying/victimization’ (−.09 to −.24). The correlation between school belonging and school supportiveness (.59) was significantly higher than any other in this domain.

#### *Physical Health and Well*-*Being and Constructive Use of Time After School*

Given that the MDI’s Physical health and well-being domain and the Constructive use of time after school domain consists of individual items, not scales, no factor analyses were conducted. In validating that the MDI scales/items represent latent constructs, the focus here is on correlations between the overall health item and indicators of children’s nutrition and sleep habits. ‘Overall health’ correlated positively with frequency of breakfast (*r* = .09), meals with family members (*r* = .14), (subjective) sleep quality (*r* = .25), and negatively with bedtime (*r* = −.08; i.e., children going to bed later reported lower overall health).

## Discussion

The MDI initiative is of particular scholarly and practical significance because of several unique features. First, the MDI presents, to our knowledge, the only initiative to collect and map data at a population-level on social and emotional development, connectedness, physical health and well-being, school experiences, and after school time use, for children during middle childhood (cf. Guhn et al. 2012). Given the representativeness of the database, findings from the MDI have the potential to make significant contributions to the research literature on the relationship between children’s social and emotional development, health, and well-being, and the presence of contextual assets in children’s most significant social contexts (family, school, community). Second, MDI data are collected in a manner that allows individual linkage to population-level databases containing longitudinal information on children’s developmental status. Currently, MDI grade 4 data are being linked to developmental health data in kindergarten (collected via the Early Development Instrument [EDI]; Janus and Offord 2007) and academic achievement data in grade 4 (collected via provincial standardized tests). Such population-level data on developmental trajectories has the potential to provide unbiased output according to socioeconomic, administrative, geographic, ethnic, gender and risk factor subpopulations; making it of particular value for researchers, as well as practitioners and policy makers (cf. Roos et al. 1995; www.childdevelopmentmonitoring.net/).

Finally, the MDI has unique potential to contribute to knowledge mobilization and translation of research evidence into educational practices in schools and communities (cf. Guhn et al. 2012; Shonkoff and Bales 2011). Collaboration between university, school district, and community organization ensures that the data are both scientifically rigorous and of immediate relevance for community development, educational practice and policy. MDI data are shared with schools via individual school reports, and with communities via community reports and community mapping (earlylearning.ubc.ca/mdi). The fact that MDI are representative at the local level allows schools to evaluate their own results in regard to district averages, and allows communities to compare their own results to those of other communities. Accordingly, schools, districts, and community organizations use MDI results to inform their program planning (e.g., Vancouver School Board 2010; School District 19 2012).

### Psychometric Findings

The psychometric results presented here indicate strong psychometric reliability and validity of the MDI in our samples. Cronbach’s alphas for the MDI scales ranged from .65 to .87, and ordinal alphas from .70 to .91. High alphas were expected insofar as most MDI items and scales were drawn from measures that have been widely used and psychometrically examined in human development research. For example, the Satisfaction with Life Scale adapted for Children (SWLS-C; Gadermann et al. 2010, 2011) has shown high internal consistency and theoretically coherent convergent and discriminant validity patterns, and think-aloud protocols with children showed that children’s responses to the SWLS-C items are consistent with theoretical frameworks on satisfaction with life.

The exploratory factor analyses in Study 3 indicated that almost all items had the highest factor loadings on the factors corresponding to their respective MDI scales. The one exception occurred for the items on the self-concept scale, as two of the three scale items had relatively high cross-loadings with the satisfaction with life scale. However, after revisions of the response format and the order of items on the MDI, the confirmatory factor analyses in Study 4 indicated excellent model fit for all scales, including the self-concept scale. Finally, the correlation patterns between subscales within the MDI domains indicate high convergent and discriminant validity of the MDI scales. For example, as expected, the scales for optimism, self-concept, and satisfaction with life correlated strongly positively with each other, and negatively with sadness and worries, whereas the scales of empathy and prosocial behavior exhibited correlations of small effect size with sadness and worries, and small to moderate ones with optimism, self-concept, and satisfaction with life. These findings are in line with previous research findings (Gadermann et al. 2010; Huebner and Alderman 1993; Lucas et al. 1996). Thus, our findings indicate that children at age 10 clearly, and in theoretically predictable ways, differentiate between positive and negative aspects of their well-being.

Our psychometric findings indicate that children at age 10 understand and interpret the MDI items in ways that are compatible with psychological theories and previous findings. We do, however, acknowledge that our study presents solely the first step in a comprehensive validation research program. Given that the MDI is designed to be used at population levels in Canada as well as in other countries—for example, Australia is piloting the MDI at population levels in 2013—validation research needs to examine for what purposes the MDI can be used adequately. At least equally important, the MDI validation research program needs to determine the limits of usage, to avoid that the MDI is used for purposes for which it is not valid. The following section briefly delineates what future validation studies are currently planned for the MDI.

### Future Validation Research

Future validation research with the MDI needs to explore to what extent children’s responses on MDI scales (1) can be predicted by developmental health outcomes at an earlier age; (2) have concurrent validity in regard to developmental health outcomes not measured on the MDI; (3) show inter-rater reliability when compared to parent or teacher ratings; and (4) demonstrate predictive validity in regard to children’s and adolescents’ later developmental health outcomes. Apart from correlational studies that link MDI data to other measures, future studies also need to explore to what extent the MDI measures constructs similarly for different subpopulations. Studies need to employ multi-group confirmatory factor analyses and differential item functioning to test whether measurement models are equivalent across subgroups (e.g., gender) and whether individual items or scales are biased against certain subgroups. Furthermore, we recommend that researchers conduct think-aloud studies to examine children’s reasoning behind their responses. Last—and most important—validation research pertaining to the MDI needs to critically examine which inferences and decisions can be drawn validly based on MDI data. That is, the validation of a measure like the MDI, which explicitly aims to foster practices and decisions in support for children, as a consequence of the measurement process, requires that this aspect be deliberately evaluated (cf. Guhn et al. 2011).

Theoretically, the MDI is rooted in developmental theory. More broadly speaking, however, the MDI is conceptualized within the Social Indicators approach as well as the Rights of the Child framework, in that the MDI partly fulfills children’s ‘right to express their views in all matters affecting them’ (Article 12 of the UN Convention on the Rights of the Child; www.unicef.org/crc/). Whether the MDI will accomplish its purpose will therefore have to be evaluated by monitoring to what extent the MDI creates awareness and opportunities for issues that reflect the primary concerns for children’s physical and mental health and well-being.

## Conclusion

In regard to research and public interest, the MDI appears to fill a niche, by integrating methods and theoretical concepts from applied developmental research, social indicators research, and population health, and by focusing on middle childhood. Whereas there is a relative abundance of research on adolescent health and well-being, and, especially during the past decades, on the ‘importance of the early years’, there has been a relative lack of research on children’s developmental health and well-being during middle childhood. In particular, population-level studies that capture children’s developmental health and well-being as well as associated social and contextual assets from the child perspective, and at representative population levels do not exist—despite the fact that middle childhood represents a developmental period that is particularly apt for prevention and intervention efforts that target actionable factors in children’s multiple environments: social relationships at home, school and community; sleep and nutrition habits; school experiences; and after-school time use. What remains to be seen is to what extent the MDI can fulfill its promise to prompt action in schools and communities, and whether these actions will benefit the mental and physical health and well-being of children and their families.

## Footnotes

1 This article focuses on the MDI development and validation, and not on school- or neighborhood-level aggregation of data, neighborhood-level linkage to socio-economic status data, or longitudinal, child-level linkage to data on earlier or later education or health outcomes. We provide this information, however, because building in the possibility for data linkages at population-level has been an integral part of the MDI project and its knowledge-to-action purpose.

2 Two domains on the MDI—physical health and well-being; after-school time—solely use single item measures, and no scales.

3 Please refer to Table 3. Also, some of the associations of subscales across domains (examining the associations between developmental outcomes and social and contextual assets) are reported elsewhere (Guhn et al. 2012). All other correlations are available upon request from the authors.

4 All reported comparisons in this result section were significant at the .05 level.
